# Outcomes following coronary artery bypass grafting with temporary left ventricular assist device support

**DOI:** 10.3389/fcvm.2026.1842278

**Published:** 2026-05-21

**Authors:** Duc M. Giao, Adham Makarem, Ameen M. Basha, Chijioke Chukwudi, Ruby Singh, Boateng Kubi, George E. Olverson, Thais Faggion Vinholo, Aabha Divya, Kamila Drezek, David A. D’Alessandro, Eriberto Michel, Asishana A. Osho

**Affiliations:** 1 Division of Cardiac Surgery, Corrigan Minehan Heart Center, Massachusetts General Hospital, Harvard Medical School, Boston, MA, United States; 2 Department of Cardiac Surgery, Smidt Heart Institute, Cedars Sinai Medical Center, Los Angeles, CA, United States; 3 Division of Cardiac Surgery, University of Calgary, Calgary, AB, Canada; 4 Division of Cardiac Surgery, Brigham and Women’s Hospital, Harvard Medical School, Boston, MA, United States

**Keywords:** CABG, high-risk cardiac surgery, impella, LVAD, mechanical circulatory support (MCS)

## Abstract

**Background:**

The Impella 5.5 is a temporary mechanical circulatory support device consisting of a microaxial pump that unloads the left ventricle and provides hemodynamic support, but data in bypass grafting (CABG) are limited.

**Methods:**

We conducted a single-center retrospective case series of adult patients who underwent isolated CABG with perioperative Impella 5.5 support between July 2020 and May 2024. Perioperative support was defined as implantation before separation from cardiopulmonary bypass (CPB) or immediately after CPB wean. Patients with prior mechanical circulatory support, preoperative Impella as the primary indication, or concomitant/reoperative cardiac surgery were excluded. Clinical characteristics, procedural details, and early outcomes were obtained from the electronic medical record.

**Results:**

Sixteen patients met inclusion criteria. Median age was 59 years (range: 51–90 years), 93.8% were male, and 75.0% self-identified as White. Median preoperative LVEF was 20.5% (IQR: 15–25). Median Impella support duration was 6.97 days (IQR: 3.7–20.3 days). The device was implanted before CPB separation in 8 patients (50.0%) and immediately after CPB wean in 8 (50.0%); 93.8% were implanted via a right axillary graft. Thirteen patients (81.3%) were successfully weaned, and 1 patient (6.3%) was bridged to HeartMate 3. Two patients (12.5%) died before device explantation, and 1 additional patient died later during the same hospitalization after explantation, yielding an overall in-hospital mortality of 3 patients (18.8%). Rhythm complications occurred in 9 patients (56.3%), most commonly rapid atrial fibrillation (31.3%); sustained ventricular tachycardia occurred in 2 (12.5%), with 1 death. One intrapump thrombus required device exchange; no major bleeding or access-site infections occurred.

**Conclusion:**

Impella 5.5 support in high-risk CABG with LV dysfunction was feasible in selected patients and may serve as an adjunct to surgical revascularization.

## Key takeaways

- Perioperative Impella 5.5 assisted CABG in severe LV dysfunction was feasible, with 81% (13/16 patients) successfully weaned and one bridged to durable LVAD therapy.
- Despite high-risk features and prolonged ICU/hospital stays, device-related complications were uncommon; arrhythmias were frequent but generally manageable.
- Early mortality was 18.8% (3/16 patients), suggesting acceptable short-term outcomes and supporting Impella 5.5 as an adjunct for carefully selected high-risk CABG patients.

## Introduction

Temporary mechanical circulatory support (MCS) has emerged as an important adjunct for high-risk cardiac interventions in patients with complex coronary artery disease, severely reduced left ventricular ejection fraction (LVEF), and substantial comorbid burden (1). Among available devices, the Impella® 5.5 (Abiomed, Danvers, MA) has been adopted in both percutaneous coronary intervention (PCI) and surgical revascularization settings for patients at prohibitive procedural risk. The device is a temporary mechanical circulatory support device consisting of a microaxial pump that unloads the left ventricle by drawing blood from the LV and delivering it into the ascending aorta across the aortic valve (1).

For patients with multivessel or anatomically complex coronary artery disease, coronary artery bypass grafting (CABG) remains the standard revascularization strategy and is associated with superior long-term outcomes compared with PCI in appropriately selected patients (1–4). However, performing CABG in the setting of advanced left ventricular dysfunction and hemodynamic instability is challenging and carries substantial perioperative risk (2–4). The use of Impella support in this context has been described primarily in isolated case reports and small series, including patients with low ejection fraction undergoing on-pump and off-pump CABG and those with acute myocardial infarction complicated by cardiogenic shock. These reports suggest that temporary left ventricular (LV) unloading and hemodynamic support may facilitate surgical revascularization, but data remain limited and heterogeneous, and the optimal role and timing of Impella 5.5 support in relation to CABG are not well defined (2–4).

In this case series, we sought to describe our institutional experience using the Impella 5.5 as temporary left ventricular support in patients undergoing CABG. Specifically, we report patient characteristics, perioperative management strategies, and early postoperative outcomes in patients who received Impella 5.5 support during CABG, with the aim of informing feasibility, safety, and potential indications for this strategy in high-risk surgical revascularization.

## Methods

### Patient selection

This was a single-center retrospective case series of adult patients who underwent CABG with perioperative Impella 5.5 support between July 2020 and May 2024. Patients were identified by review of the institutional electronic medical record. Perioperative Impella support was defined as placement of an Impella 5.5 device before separation from cardiopulmonary bypass (CPB; ie. planned in advance due to low patient ejection fraction) or immediately after CPB wean (ie. failure to wean). Decision for Impella placement due to the inability to wean from cardiopulmonary bypass was done after thorough evaluation and optimization of reversible causes including volume status, rhythm issues, electrolytes, acid-base status, and residual air. Despite optimization, patients who required escalation of inotropes and pressor support to maintain mean arterial pressure (MAP) > 65, and/or the LV continued poor function identified on transesophageal echocardiography were selected for Impella placement. Patients who were supported with another mechanical circulatory support device prior to Impella insertion were excluded. We also excluded patients whose primary indication for Impella placement was preoperative optimization prior to CABG rather than intraoperative or immediate post-CPB support. In addition, patients undergoing concomitant cardiac procedures or reoperative cardiac surgery were excluded in order to focus on outcomes following isolated, first-time CABG with temporary left ventricular support. Clinical, operative, and outcome data were abstracted retrospectively from electronic medical records. During the study period, there were no formal institutional criteria for perioperative Impella 5.5 use; candidacy and timing of implantation were determined on a case-by-case basis by the attending surgeon and operative team. This study was approved by the Massachusetts General Brigham Institutional Review Board.

### Surgical technique

Selection for Impella 5.5 support and the timing of implantation were based on clinical judgment, including severe preoperative ventricular dysfunction, intraoperative hemodynamic performance, transesophageal echocardiographic assessment of ventricular function, and concern regarding the ability to separate from cardiopulmonary bypass or maintain adequate post-bypass circulation. In some patients, Impella support was planned before CPB separation because of anticipated difficulty weaning given the severity of left ventricular dysfunction and overall operative risk, whereas in others the device was placed after CPB wean in response to hemodynamic instability or inadequate ventricular performance.

All procedures were performed under general anesthesia via median sternotomy. The Impella 5.5 (Abiomed, Danvers, MA) was implanted using a surgical transthoracic approach, most commonly through a graft sewn to the right axillary artery, and less commonly through direct ascending aortic graft access. Standard institutional protocols for CABG with systemic heparinization to achieve an activated clotting time greater than 480 s and del Nido cardioplegia used for myocardial protection and perioperative hemodynamic management were followed in all cases. Vascular access was obtained by sewing an 8–10 mm vascular graft to either the right axillary artery (*n* = 15) or the ascending aorta (*n* = 1), which then permitted advancement of an appropriate vascular sheath into the graft secured at multiple points to the chest wall and skin to minimize tension and prevent displacement. The Impella 5.5 catheter was inserted over a guidewire and advanced across the aortic valve into the left ventricle under fluoroscopic and transthoracic or transesophageal echocardiographic guidance. After an initial period at low support, device speed was titrated as needed to provide up to 5.5 L/min of forward flow according to hemodynamic requirements. At the conclusion of device positioning, the vascular graft was shortened as needed, and the graft–catheter complex was secured at multiple points to the chest wall and skin to minimize tension and prevent displacement. Postoperatively, a heparin drip was started within 4–6 h after surgery provided there were no concerns of bleeding and either heparin purge or sodium bicarbonate was used for Impella purge solution (institutional protocol changed in 2021 from using heparin to sodium bicarbonate due to concerns of bleeding in patients already on systemic heparin drip). Patients were extubated within 4–6 h after the operation and ambulated three times daily.

### Outcomes

The primary outcome was in-hospital mortality following CABG. Secondary outcomes included successful weaning from Impella support or transition to a durable left ventricular assist device (LVAD), as well as major device- or rhythm-related complications identified during the index hospitalization. Rhythm-related complications were identified by retrospective review of operative reports, intensive care unit documentation, telemetry records, and inpatient progress notes and included atrial and ventricular arrhythmias occurring after Impella implantation. Device-related complications included pump thrombosis or pump malfunction requiring intervention or device exchange. Bleeding events were not prospectively adjudicated using a standardized definition; however, clinically evident major bleeding requiring reoperation, transfusion escalation, or procedural intervention was recorded when documented in the medical record. Outcomes were determined by review of the inpatient course and follow-up documentation in the electronic medical record.

### Statistics

Given the descriptive nature of this case series, analyses were limited to summary statistics. Continuous variables were summarized using medians and interquartile ranges, with calculation of a sample standard deviations where appropriate. Categorical variables were reported as counts with corresponding percentages. Outcomes were described as proportions of the overall cohort. No formal hypothesis testing was performed.

## Results

Between July 2020 and May 2024, 16 patients underwent isolated CABG with perioperative Impella 5.5 support and met inclusion criteria. Baseline characteristics are summarized in Table 1. The median age was 59 years (range: 51–90 years), and most patients were male (15/16, 93.8%). Race was documented for the majority of patients; overall, 12 (75.0%) self-identified as White. Acute coronary syndrome (ACS) was present at admission in 6 patients (37.5%), with a mean interval from ACS to CABG of 10.67 ± 8.43 days [median 8 (IQR: 7–11)]. The mean preoperative left ventricular ejection fraction was 21.2% (SD: 5.9; range: 13%–30%).

**Table 1:** Preoperative patient demographics and baseline cardiac function measurements. .

Patient ID	Age	Sex	Cardiac comorbidities	LVEF (%)	LVEDD (mm)	Vasopressors	STS risk score
1	57	M	DM, HTN, CLD, PVD	21	59	None	NA
2	75	M	HTN, CLD, PVD, CVD	23	52	None	0.04223
3	55	M	HTN, CLD, CVD	20	NA	None	0.03157
4	51	M	DM, HTN, CLD	14	58	None	0.00929
5	66	M	DM, HTN, CLD	15	NA	None	0.01868
6	68	M	DM, HTN, CLD, PVD, CVD	14	92	NA	0.20007
7	56	M	CLD	20	60	None	NA
8	59	M	HTN, CLD, CVD	25	68	None	0.02501
9	59	M	DM, CLD,	25	57	None	0.0055
10	66	M	DM, HTN, CLD, CVD	29	51	None	0.02955
11	59	M	HTN, PVD	20	NA	None	0.2018
12	54	M	DM, HTN, CLD	13	77	None	0.00863
13	73	M	HTN, CLD, CVD	30	56	None	NA
14	56	M	DM, HTN, CLD	15	NA	None	NA
15	90	F	CLD	30	57	Dobutamine	NA
16	72	M	DM, HTN, CLD	25	52	None	NA

LVEF, left ventricular ejection fraction; LVEDD, left ventricular end diastolic dimension; HTN, hypertension; CLD, chronic lung disease; DM, diabetes mellitus; PVD, peripheral vascular disease; CVD, cerebrovascular disease; NA, not available.

Procedural characteristics and device-related outcomes are shown in Table 2. The median duration of Impella support was 6.97 days (IQR: 6.1–12.8). In 8 patients (50.0%), the Impella 5.5 was inserted before separation from CPB, and in 8 patients (50.0%) it was placed immediately after CPB wean due to failure to wean from CPB. Fifteen patients (93.8%) had the device implanted via a right axillary artery graft, whereas 1 patient (6.3%) had direct ascending aortic cannulation. Heparin-based purge solution was used in 9 patients (56.3%), while 7 patients (43.8%) received sodium bicarbonate purge. One patient experienced a pump malfunction due to intrapump thrombus, necessitating exchange to a second Impella 5.5, after which no further device-related events occurred.

**Table 2:** Patient procedural data and outcomes.

ID	Duration support (days)	Procedure (CABG x)	Cross-clamp time (min)	CPB time (min)	Impella insertion timing (before/after CPB)	Access approach (R axillary vs. direct aorta)	Outcome
1	14.04	4	82	108	After	Axillary	Successfully Weaned
2	5.59	3	105	290	Before	Direct Aorta	Successfully Weaned
3	5.77	4	86	175	After	Axillary	HM3
4	6.94	4	74	108	After	Axillary	Successfully Weaned
5	20.30	5	84	100	After	Axillary	Successfully Weaned
6	7.00	3	78	97	After	Axillary	Death after Impella explantation
7	5.96	4	110	163	Before	Axillary	Successfully Weaned
8	8.12	2	64	132	Before	Axillary	Death prior to Impella explantation
9	6.14	3	113	180	Before	Axillary	Successfully Weaned
10	8.79	3	99	164	Before	Axillary	Successfully Weaned
11	6.67	3	68	118	Before	Axillary	Successfully Weaned
12	12.74	4	98	129	After	Axillary	Successfully Weaned
13	13.87	2	100	175	Before	Axillary	Successfully Weaned
14	13.17	2	53	105	Before	Axillary	Successfully Weaned
15	3.74	1	NA	NA	After	Axillary	Death prior to Impella explantation
16	6.84	3	74	117	After	Axillary	Successfully Weaned

NA, not available; CABG, coronary artery bypass grafting; CPB, cardiopulmonary bypass.

The cohort had a median preoperative length of stay of 6 days (IQR: 1.5–9.5). Postoperatively, patients required prolonged critical care support, with median ICU length of stay of 11 days (IQR: 8–16.5) and a median total postoperative hospital length of stay of 18 days (IQR: 13.5–21). Thirteen patients (81.3%) were successfully weaned from Impella support, and 1 patient (6.3%) was transitioned to a durable HeartMate 3 LVAD. Of the 8 patients with pre-CPB-separation placement, 7 survived; of the 8 placed after CPB wean, 7 survived. Two patients (12.5%) died before device explantation. One death was attributed to refractory cardiogenic shock with progressive multiorgan failure, and the other to ventricular fibrillation. All remaining patients who had the device explanted survived except for one patient who died due to multi-organ failure secondary to septic shock from serratia bacteremia, serratia and klebsiella pneumonia, and enterococcus faecium UTI. This resulted in an overall in-hospital mortality of 3 patients (18.8%). For the patients that survived to device explantation, one patient's hospital course was notable for Moraxella catarrhalis pneumonia and another patient's course was notable from a groin hematoma from CPB cannulation site. No other major complications were noted in this group.

Rhythm-related complications occurred in 9 patients (56.3%) and were predominantly transient. The most frequent arrhythmia was rapid atrial fibrillation, observed in 5 patients (31.3%) and managed medically. Ventricular ectopy developed in 2 patients (12.5%) and resolved after Impella repositioning. Sustained ventricular tachycardia occurred in 2 patients (12.5%), including the patient who died of ventricular fibrillation. No clinically documented major bleeding complications or access-site wound infections were observed during the index hospitalization.

## Discussion

In this single-center case series, we describe our experience using the Impella 5.5 for perioperative hemodynamic support in 16 high-risk patients undergoing isolated CABG with severely depressed left ventricular function [median preoperative LVEF 20.5% (IQR: 15–25)]. Our findings suggest that Impella 5.5 support is feasible in selected high-risk patients undergoing CABG, with most patients either recovering sufficient native cardiac function or being successfully bridged to durable LVAD therapy. Of the 13 patients who survived to Impella 5.5 explantation, one eventually died due to multi-organ failure.

Prior early U.S. experience with the Impella 5.5 across a range of indications demonstrated survival to explant in approximately 80% of patients and high rates of native heart recovery (2). Additional reports have described its use in complex coronary revascularization and cardiogenic shock, including off-pump CABG in the setting of post–myocardial infarction shock (3). Our data are broadly consistent with these observations. In our cohort, 13 patients (81.3%) were successfully weaned from Impella support and 1 patient (6.3%) was transitioned to a durable HeartMate 3 LVAD, indicating that temporary LV unloading with the Impella 5.5 can facilitate both myocardial recovery and structured transition to advanced heart failure therapies in appropriately selected CABG patients.

The timing of mechanical circulatory support initiation is increasingly recognized as a key determinant of outcomes in post-cardiotomy shock (5, 6). Studies comparing early (intraoperative or prophylactic) vs. delayed Impella implantation have reported superior short- and mid-term survival when the device is placed at the time of CABG rather than as a rescue strategy for established cardiogenic shock (6). In our series, all patients received Impella 5.5 support intraoperatively, either before separation from CPB (50%) or immediately after CPB wean (50%). This approach likely contributed to hemodynamic stability during the vulnerable peri-weaning period, allowing for gradual ventricular unloading and recovery following surgical revascularization. Although our sample is small and non-comparative, these findings align with the growing body of evidence favoring earlier, planned MCS deployment in high-risk surgical candidates rather than delayed rescue implantation.

Arrhythmias were common but generally manageable in our cohort. Overall, rhythm-related complications occurred in 9 patients (56.3%). The most frequent arrhythmia was rapid atrial fibrillation, observed in 5 patients (31.3%) and controlled medically. Ventricular ectopy occurred in 2 patients (12.5%) and resolved after Impella repositioning, supporting a mechanistic link between intracavitary device position and ectopic activity. Sustained ventricular tachycardia developed in 2 patients (12.5%), with one death due to ventricular fibrillation. These observations corroborate prior reports of a high incidence of arrhythmias in Impella-supported patients, often without a clear associated increase in mortality when promptly recognized and treated (7). Our experience underscores the importance of vigilant rhythm surveillance and expert device management, including early consideration of device repositioning when ectopy emerges. We theorize that high Impella flows can decrease LV cavity size and precipitate ventricular arrhythmias as the pump contacts the endocardium; in such cases in particular, using transthoracic echocardiography to monitor the position of Impella and contractility of the LV is critical.

Device-related complications were infrequent. One patient experienced pump malfunction due to intrapump thrombus, necessitating exchange to a second Impella 5.5 without further adverse events. Notably, we did not observe clinically significant bleeding events or access-site infections despite a median support duration of approximately 7 days (range: 3.7–20.3 days). Compared with earlier generations of axial-flow pumps, the design modifications of the Impella 5.5, including elimination of the pigtail and improvements in catheter robustness, may contribute to its safety profile during extended support (2–6, 8). Nevertheless, isolated reports have described device-related complications at explant, such as valvular injury or device fracture (9, 10). The absence of such events in our series is reassuring but should be interpreted with caution given the limited sample size.

### Limitations

This study has few limitations. First, the sample size is relatively small (*n* = 16), which limits statistical precision and precludes robust conclusions regarding prognostic factors or comparative effectiveness vs. other forms of MCS or conventional management. Second, this is a retrospective, single-center experience, and our patient selection, operative techniques, and perioperative management protocols may not be generalizable to other institutions. Third, we did not include a control group of similarly high-risk CABG patients managed without Impella support or with alternative devices. Accordingly, this study cannot estimate the incremental benefit, comparative effectiveness, or causal impact of Impella 5.5 support on postoperative outcomes. Additionally, the decision to implant Impella 5.5 and the timing of support were not standardized, introducing potential selection bias and limiting reproducibility. Lastly, some data regarding left ventricular end diastolic dimension (LVEDD) as reported in Table 2 were missing due to suboptimal transthoracic echocardiographic windows, which limited accurate visualization for linear LV measurements and some data regarding STS Risk scores were missing due to incomplete capture of required preoperative variables in the retrospective record, particularly among urgent or emergent cases where full data entry was not consistently documented.

Future studies should define objective clinical, echocardiographic, and hemodynamic criteria for perioperative Impella use in high-risk CABG. Finally, follow-up was limited to the index postoperative hospitalization following CABG. Accordingly, longer-term outcomes after discharge, including survival, rehospitalization, ventricular recovery, and quality of life, were not systematically captured. Larger, multicenter studies with standardized indications, timing strategies, and follow-up are needed to more definitively characterize the role of Impella 5.5 in high-risk CABG.

## Conclusion

In this single-center case series of high-risk patients undergoing CABG with severely reduced LVEF, perioperative support with the Impella 5.5 was feasible in selected patients. Our institutional experience suggests that Impella 5.5 can serve as a valuable adjunct to maintain hemodynamic stability and facilitate myocardial recovery or structured bridging in selected high-risk CABG patients. Future larger-scale studies are warranted to refine patient selection, optimize timing of implantation, and define the comparative effectiveness of this strategy within the broader landscape of perioperative mechanical circulatory support.

## Data Availability

The original contributions presented in the study are included in the article/Supplementary Material, further inquiries can be directed to the corresponding author.
